# Prognostic Significance of Peri-Treatment Inflammatory Burden Index in Patients with Rectal Cancer Undergoing Preoperative Chemoradiotherapy

**DOI:** 10.3390/cancers18172784

**Published:** 2026-08-27

**Authors:** Takanobu Mori, Yoshinaga Okugawa, Tadanobu Shimura, Ma Ruiya, Naru Mizuno, Shinji Yamashita, Akira Yamamoto, Takahito Kitajima, Hiroki Imaoka, Mikio Kawamura, Yuhki Koike, Hiromi Yasuda, Yoshiki Okita, Shigeyuki Yoshiyama, Minako Kobayashi, Tomofumi Koide, Masaki Ohi, Yuji Toiyama

**Affiliations:** 1 Department of Clinical Nutrition, Mie University Hospital, Tsu 514-8507, Mie, Japan; t-mori-n@med.mie-u.ac.jp (T.M.);; 2 Department of Gastrointestinal and Pediatric Surgery, Division of Reparative Medicine, Institute of Life Sciences, Mie University Graduate School of Medicine, Tsu 514-8507, Mie, Japanshinji-y@med.mie-u.ac.jp (S.Y.); t-kitajima@med.mie-u.ac.jp (T.K.); h-imaoka@med.mie-u.ac.jp (H.I.);; 3 Department of Genomic Medicine, Mie University Hospital, Tsu 514-8507, Mie, Japan

**Keywords:** rectal cancer, inflammatory burden index, chemoradiotherapy, recurrence, prognosis

## Abstract

**Background/Objectives:** Systemic inflammatory responses influence treatment response and oncological outcomes in patients with cancer. Growing evidence indicates that the inflammatory burden index (IBI) serves as a reliable prognostic indicator across various malignancies. However, the clinical significance of the peri-treatment inflammatory status in patients with rectal cancer (RC) undergoing preoperative chemoradiotherapy (CRT) remains unclear. **Methods:** We assessed the pre- and post-treatment IBI in 97 patients with RC who received preoperative CRT followed by total mesorectal excision at our institution. **Results:** Although no significant changes were observed in the pre- or post-CRT IBI and no associations were found with most clinicopathological factors, survival analysis revealed that a high pre-CRT IBI was significantly associated with shorter overall survival (OS) and disease-free survival (DFS). Multivariable analysis revealed that a high pre-CRT IBI and pathological lymph node metastasis remained independent prognostic factors for both outcomes. Subgroup analysis demonstrated that the prognostic value of the peri-treatment IBI differed according to the ypN status, with the pre-CRT IBI showing particularly strong prognostic performance in patients without pathological lymph node metastasis (ypN−). **Conclusions:** Pre-treatment assessment of the IBI may improve risk stratification in patients with RC undergoing preoperative CRT followed by curative resection.

## Simple Summary

Even when their cancer appears similar after treatment, patients with rectal cancer who receive chemotherapy and radiation therapy before surgery can have different long-term outcomes. Therefore, identifying simple ways to recognize patients at higher risk of poor outcomes could help to improve their care after surgery. In this study, we examined the inflammatory burden index, which is calculated using results from routine blood tests and reflects inflammation in the body. We found that patients with a high inflammatory burden index before treatment had poorer long-term outcomes than those with a low index. This was also observed among patients whose lymph nodes showed no signs of cancer after treatment. These findings suggest that the inflammatory burden index could provide additional information about long-term outcomes and help identify patients who may need closer follow-up after surgery.

## 1. Introduction

Colorectal cancer (CRC) is one of the most commonly diagnosed malignancies worldwide and is the second leading cause of cancer-related mortality in Western countries [1]. In rectal cancer (RC), local recurrence has historically been a major determinant of oncological outcomes. Preoperative chemoradiotherapy (CRT) followed by total mesorectal excision (TME) is the standard treatment for locally advanced RC and has been shown to improve sphincter preservation, local control, and overall survival (OS) [2]. However, distant recurrence still occurs in approximately 20% of patients, and pathological response to CRT is strongly associated with oncological outcomes [3,4]. Therefore, reliable biomarkers are needed to identify which patients with RC receiving preoperative CRT are most likely to benefit in terms of survival outcomes and which may require closer postoperative surveillance for the early detection of recurrence.

Systemic inflammation is a recognized hallmark of cancer that drives disease progression, treatment resistance, and poor survival [4]. These inflammatory responses promote tumor growth, angiogenesis, and metastasis while simultaneously modulating the host immune response [5,6,7,8]. Increasing evidence suggests that the interaction between tumor biology and host inflammatory status influences treatment response and long-term outcomes in patients with cancer, including CRC [9]. Consequently, several inflammation-based indices have been proposed as clinically useful biomarkers of systemic inflammatory activity and host immune status.

Among these indices, the inflammatory burden index (IBI) is a practical composite biomarker calculated from routinely available laboratory parameters, including C-reactive protein (CRP), neutrophil count, and lymphocyte count [10]. These parameters reflect systemic inflammatory activity and host immune status, both of which are closely associated with oncological outcomes. Previous studies have demonstrated the prognostic value of the preoperative IBI in several cancer types, including esophageal [11], hepatocellular [12], colorectal [13], gastric [14], bladder [15], and lung cancers [16]. However, whether the peri-treatment IBI during neoadjuvant CRT predicts long-term oncological outcomes in patients with RC remains unclear.

We previously evaluated several inflammation-based indices as noninvasive predictors of perioperative risk and survival in patients with gastrointestinal malignancies, particularly CRC [17,18,19]. The present study aimed to evaluate the association between clinicopathological factors and the peri-treatment IBI measured before and after preoperative CRT in patients with RC undergoing TME and to assess the potential value of the IBI as a predictor of oncological outcomes in these patients.

## 2. Materials and Methods

### 2.1. Patients

This retrospective single-center analysis included 97 individuals diagnosed with RC who underwent TME subsequent to preoperative CRT from 2001 through 2015. Neoadjuvant chemoradiation was indicated for patients meeting specific baseline criteria: distal or mid-rectal lesions (lower two-thirds of the rectum); locally advanced tumors classified as clinical T2–T4, or clinical T1 displaying deep submucosal penetration with regional nodal involvement (cN1), intended to optimize R0 resectability and sphincter preservation rates; an age range of 20 to 80 years; preserved functional status (Eastern Cooperative Oncology Group performance status 0–1); and the absence of both pelvic muscular involvement (external anal sphincter or levator ani) and deep venous thrombosis.

Pretreatment clinical staging was determined using digital rectal examination, transrectal ultrasonography, computed tomography, and magnetic resonance imaging. Tumor staging was performed according to the Tumor–Node–Metastasis (TNM) classification of the International Union Against Cancer (UICC), 7th edition. Of the 97 patients, 11 were classified as having stage I disease, 16 as having stage II disease, and 70 as having stage III disease before CRT. Consent was obtained on an opt-out basis, and patients who did not wish to provide their medical information were excluded from the study. The study protocol was reviewed and approved by the institutional review board of our institution (Approval No. H2026-046).

### 2.2. 5-Fluorouracil (5-FU)–Based CRT Regimen

Radiotherapy was administered as either a short-course regimen (*n* = 44; 20 Gy delivered in four fractions) or a long-course regimen (*n* = 53; 45 Gy delivered in 25 fractions), both using a four-field box technique. Concurrent chemotherapy consisted of 5-FU–based regimens, including 5-FU in combination with leucovorin, tegafur/uracil, or S-1. The interval between completion of CRT and surgery was 2–3 weeks for patients receiving the short-course regimen and 6–8 weeks for those receiving the long-course regimen. Surgical procedures were performed using either an open or laparoscopic approach, and all patients underwent curative-intent resection based on TME principles. Following surgery, all patients received 5-FU-based adjuvant chemotherapy for 6–12 months. Postoperative monitoring adhered to our department’s standardized surveillance program, with patient visits scheduled every 12 to 16 weeks during the initial year. Routine follow-up evaluations encompassed serial serum tumor marker quantification, computed tomography scans, endoscopic examinations, abdominal ultrasonography, and plain chest radiographs. In addition, skeletal scintigraphy was selectively conducted whenever clinical findings raised suspicion of osseous dissemination.

Clinicopathological data were retrospectively collected from inpatient and outpatient medical records. Variables included sex, age, clinical T category (cT), clinical lymph node metastasis, clinical stage, histological type, pathological T category (ypT), venous invasion, lymphatic invasion, lymph node metastasis, radiation course, pathological response to CRT, and UICC stage.

### 2.3. Laboratory Measurement of IBI

Venous blood specimens were obtained from each participant at two predefined time points: within seven days prior to commencing chemoradiotherapy (pre-CRT) and within seven days prior to surgical resection after completing CRT (post-CRT). As part of routine laboratory testing, serum CRP levels and peripheral blood neutrophil and lymphocyte counts were measured. The IBI was calculated according to the following formula: IBI = CRP × (neutrophil count/lymphocyte count), as previously described [14].

### 2.4. Pathological Assessment of Tumor Regression and Staging After CRT

Surgical specimens underwent routine pathological evaluation, with anatomical staging determined per the American Joint Committee on Cancer (AJCC) TNM staging criteria. Treatment response was quantified using the histopathological tumor regression grade (TRG) based on the Japanese Society for Cancer of the Colon and Rectum guidelines, stratified into five distinct tiers: grade 0 (no recognizable regressive change or necrosis), grade 1a (viable residual tumor cells (VRTCs) exceeding two-thirds of the tumor area), grade 1b (VRTCs occupying between one-third and two-thirds), grade 2 (VRTCs constituting less than one-third), and grade 3 (complete absence of viable residual cancer cells) [20]. Patients with TRG 0–1b were classified as nonresponders, whereas those with TRG 2–3 were classified as responders.

### 2.5. Statistical Analysis

All statistical analyses were performed using MedCalc software version 23.4.8 (MedCalc Software Ltd., Ostend, Belgium). Continuous variables are presented as median with interquartile range (IQR). Between-group differences were evaluated using the Kruskal–Wallis test for continuous variables, as appropriate. Changes in the IBI between pre-CRT and post-CRT measurements within the same patients were analyzed using the Wilcoxon signed-rank test for paired samples.

Survival probabilities were estimated using the Kaplan–Meier method, and differences between groups were evaluated with the log-rank test. Receiver operating characteristic curve analysis was conducted to identify the optimal cutoff values for the survival outcome. The optimal thresholds were determined based on Youden’s index. OS was defined as the interval from the date of surgery to death from any cause or the date of last follow-up. Disease-free survival (DFS) was defined as the interval from curative resection to the first documented recurrence, death from any cause, or the date of last follow-up. Cox proportional hazards regression models were used to estimate hazard ratios (HRs) for recurrence and death. The proportional hazards assumption was evaluated by visual inspection of Kaplan–Meier survival curves, confirming that no substantial crossing of the curves was observed. Variables with *p* < 0.05 in the univariate analysis were entered into the multivariable Cox proportional hazards regression model. In addition to the primary variable of interest (the IBI status), clinical variables included in the univariate and multivariable analyses were selected as potential confounders based on their previously reported prognostic relevance in patients with RC. These variables comprised sex, age at diagnosis, histological differentiation (well to moderately differentiated vs. poorly differentiated), pathological T stage (T1–2 vs. T3–4), venous invasion (present vs. absent), lymphatic invasion (present vs. absent), lymph node metastasis (present vs. absent), and TRG following CRT. All *p* values were two-sided, and *p* < 0.05 was considered statistically significant.

## 3. Results

### 3.1. Comparison Between Peri-CRT IBI Status and Correlation with Clinicopathological Variables

**Figure 1:**
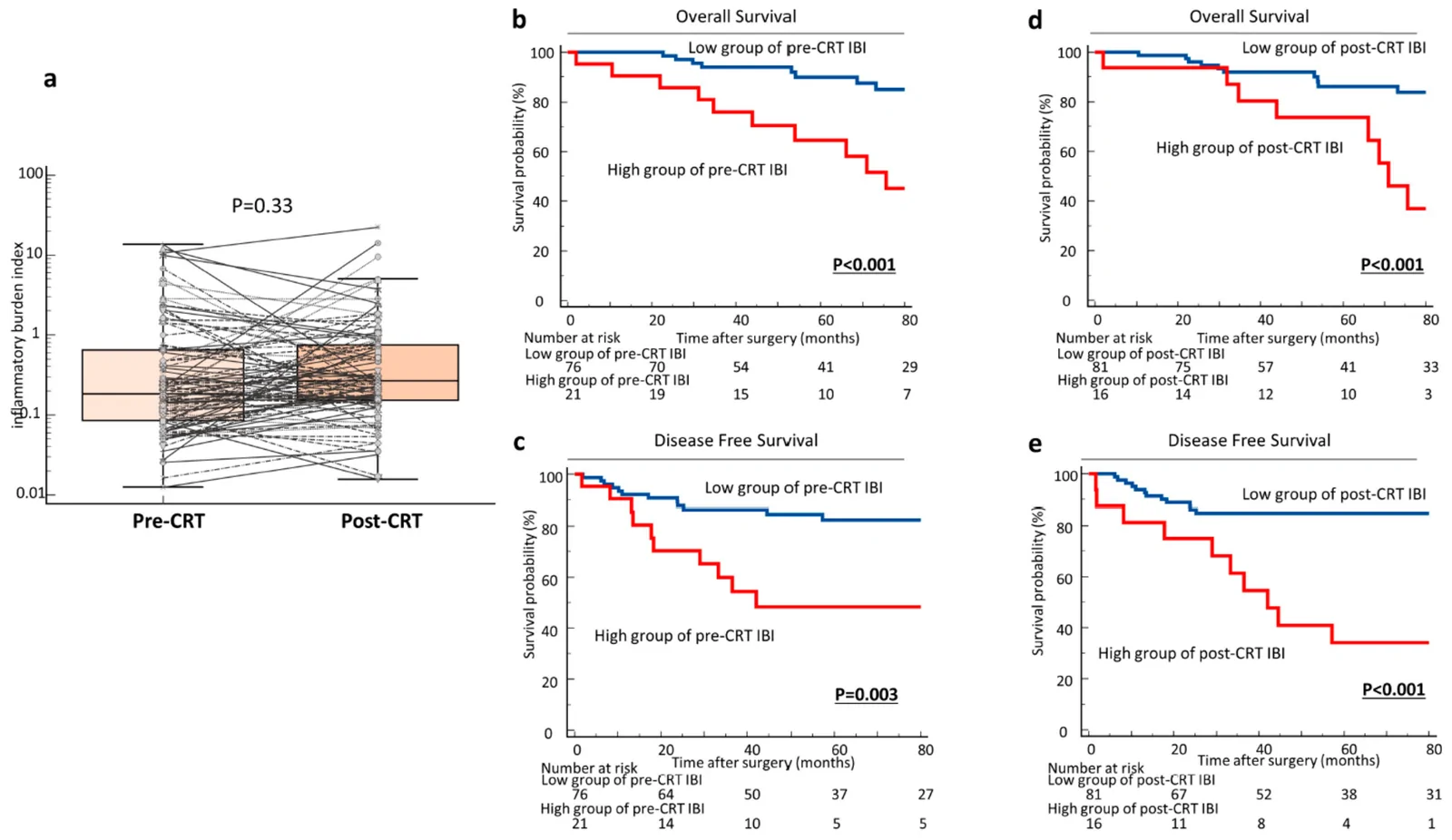
Peri-treatment changes in IBI and association with survival outcomes. (**a**) Comparison of IBI values before and after preoperative CRT. Lines connect the pre- and post-CRT IBI values for each patient. The IBI did not differ significantly between the pre-CRT and post-CRT measurements (median [IQR], 0.18 [0.08–0.64] vs. 0.27 [0.15–0.74]; *p* = 0.33; Wilcoxon signed-rank test). (**b**,**c**) Kaplan–Meier survival curves for (**b**) OS and (**c**) DFS according to the pre-CRT IBI status in patients with RC. Patients with a high pre-CRT IBI had significantly shorter OS (*p* < 0.001, log-rank test) and DFS (*p* = 0.003) than those with a low pre-CRT IBI. (**d**,**e**) Kaplan–Meier survival curves for (**d**) OS and (**e**) DFS according to the post-CRT IBI status in patients with RC. Patients with a high post-CRT IBI had significantly shorter OS (*p* < 0.001) and DFS (*p* < 0.001) than those with a low post-CRT IBI. Cut-off thresholds of pre- and post-CRT IBI were determined by ROC analysis with Youden’s index for survival outcome in this cohort. All statistical tests were two-sided.

First, we evaluated changes in the IBI before and after preoperative CRT in our cohort of patients with RC. The post-CRT IBI did not differ significantly from the pre-CRT IBI in patients with RC receiving neoadjuvant CRT (median [IQR], 0.27 [0.15–0.74] vs. 0.18 [0.08–0.64], respectively; *p* = 0.33) (Figure 1a).

Next, we analyzed the associations between the pre- and post-CRT IBI status and pre-CRT and post-operative clinicopathological variables. Nearly all clinicopathological variables were not significantly associated with either the pre- or post-CRT IBI, with the exception of sex (pre-CRT, *p* = 0.02; post-CRT, *p* = 0.04) (Table 1). Furthermore, neither pre- nor post-CRT IBI status showed a significant correlation with tumor regression grade or pathological complete response. However, a high pre-CRT IBI was significantly associated with the subsequent development of distant metastasis (*p* = 0.04). In contrast, a high post-CRT IBI was significantly correlated with positive surgical margins (*p* = 0.003).

### 3.2. Prognostic Impact of Peri-CRT IBI Status on Survival Outcomes in Patients with RC Undergoing Curative Resection After Preoperative CRT

To evaluate the prognostic significance of the IBI status measured before and after CRT, we performed time-to-event analyses using Kaplan–Meier survival curves stratified by the IBI status. Patients with a high pre-CRT or post-CRT IBI had significantly poorer OS and DFS than those with a low IBI status (log-rank test: pre-CRT, OS, *p* < 0.001 and DFS, *p* = 0.003; post-CRT, OS, *p* < 0.001 and DFS, *p* < 0.001) (Figure 1b–e).

### 3.3. High Pre-CRT IBI Status and Pathological Lymph Node Metastasis Were Independent Prognostic Factors for Both OS and DFS in Patients with RC Undergoing Curative Surgery After Neoadjuvant CRT

To further clarify the prognostic impact of the peri-CRT IBI status in patients with RC undergoing curative surgery after neoadjuvant CRT, we performed univariable Cox proportional hazards regression analyses. These analyses identified advanced pathological T stage (ypT3/4), venous invasion, pathological lymph node metastasis (ypN+), a high pre-CRT IBI, and a high post-CRT IBI as significant prognostic factors for both OS and DFS (Table 2). Multivariable Cox proportional hazards regression analyses identified a high pre-CRT IBI and pathological lymph node metastasis as independent prognostic factors for both OS and DFS (Table 2). For a high pre-CRT IBI, the hazard ratios (HRs) were 3.43 (95% CI, 1.42–8.27; *p* < 0.01) for OS and 2.73 (95% CI, 1.13–6.62; *p* = 0.03) for DFS. For pathological lymph node metastasis, the HRs were 4.76 (95% CI, 1.72–13.21; *p* < 0.01) for OS and 5.06 (95% CI, 1.87–13.7; *p* < 0.01) for DFS.

### 3.4. Prognostic Value of Dynamic Change in Pre- and Post-CRT IBI Status in Patients with RC Undergoing Curative Surgery After Preoperative CRT

We further conducted an additional analysis to clarify the dynamic changes in IBI status between the pre- and post-CRT timings in this cohort. Interestingly, evaluating IBI status changes before and after CRT may help stratify the prognosis of RC patients undergoing preoperative CRT followed by curative surgery, even though some groups are small (OS: *p* = 0.0001, DFS: *p* < 0.0001; Supplementary Figure S1a and S1b, respectively). Furthermore, multivariable Cox proportional hazards regression analyses revealed that a sustained high IBI status between the pre- and post-CRT timing (high pre-/high post-CRT IBI group) is an independent prognostic factor for both OS and DFS in this cohort (OS: HR, 8.32; 95% CI, 3.04–22.78; *p* < 0.01; DFS: HR, 8.29; 95% CI, 3.23–21.29; *p* < 0.01; Supplementary Table S1, respectively). Taken together, these findings suggest that evaluating dynamic changes in IBI status between the pre- and post-CRT timings could identify patients at high-risk for poor survival outcomes among those with RC undergoing curative surgery after preoperative CRT.

### 3.5. Prognostic Value of IBI in Patients with RC Receiving Preoperative CRT With or Without Pathological Lymph Node Metastasis

**Figure 2:**
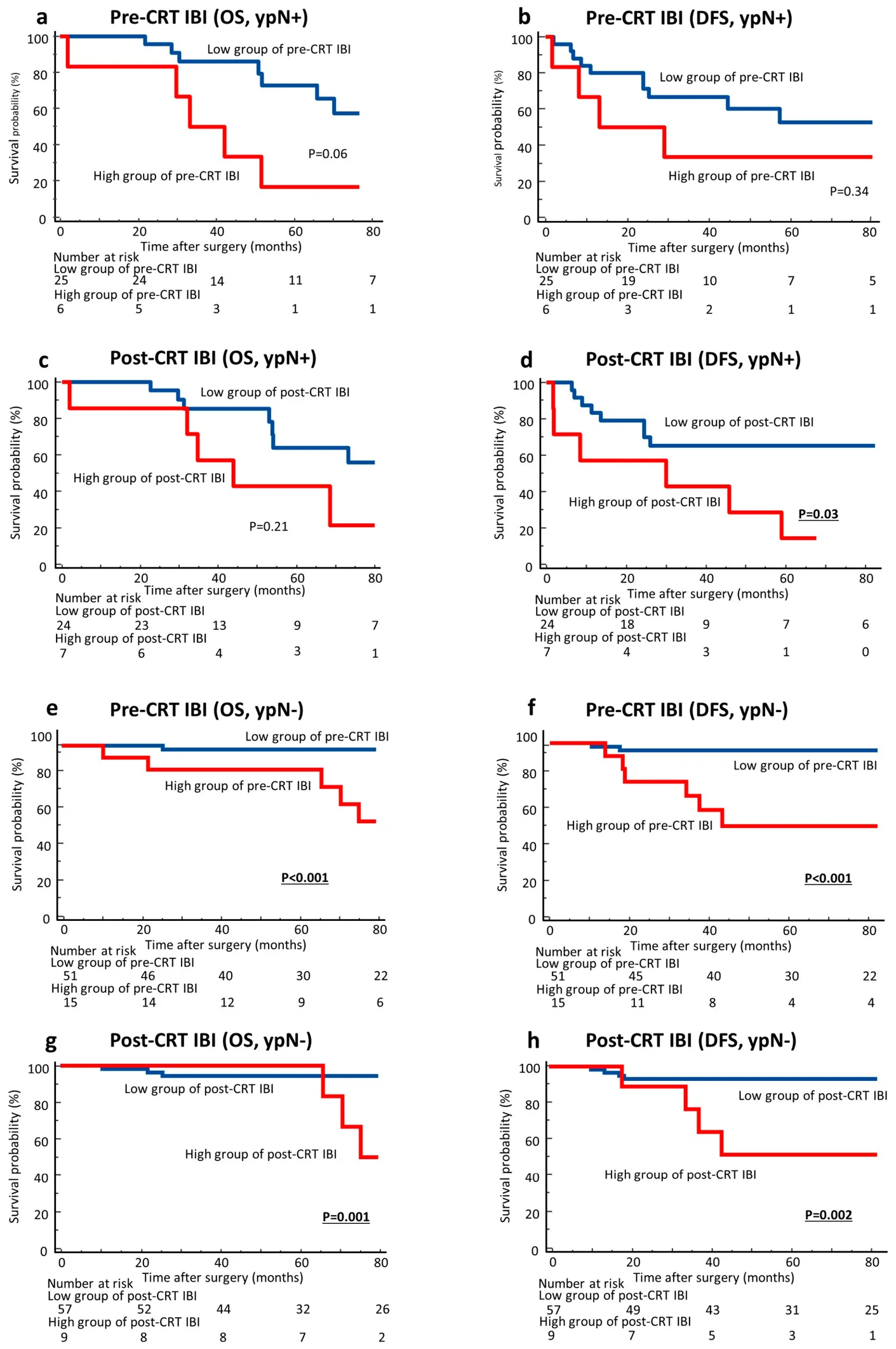
Prognostic impact of IBI stratified according to pathological lymph node metastasis. (**a**–**d**) Kaplan–Meier curves for patients with pathological lymph node metastasis (ypN+). Neither the pre- nor post-CRT IBI status was significantly associated with OS or DFS, with the exception of a significant association between a high post-CRT IBI and poorer DFS in this subgroup (log-rank test: (**a**) pre-CRT, OS, *p* = 0.06 and (**b**) DFS, *p* = 0.34; (**c**) post-CRT, OS, *p* = 0.21 and (**d**) DFS, *p* = 0.03). (**e**–**h**) Kaplan–Meier curves for patients without pathological lymph node metastasis (ypN−). Both a high pre-CRT IBI and a high post-CRT IBI were significantly associated with poorer OS and DFS than a low IBI in patients with ypN− RC (log-rank test: (**e**) pre-CRT, OS, *p* < 0.001 and (**f**) DFS, *p* < 0.001; (**g**) post-CRT, OS, *p* = 0.001 and (**h**) DFS, *p* = 0.002). Cut-off thresholds of pre- and post-CRT IBI were determined by ROC analysis with Youden’s index for survival outcome in this cohort. All statistical tests were two-sided.

The multivariable analysis described above demonstrated that pathological lymph node metastasis was a strong independent prognostic factor in patients with RC undergoing preoperative CRT. Based on these findings, we further evaluated the prognostic value of the peri-CRT IBI status according to the pathological lymph node status. We first focused on patients with RC with pathological lymph node metastasis (ypN+) and performed time-to-event analyses. Kaplan–Meier analysis showed no significant association between the pre- or post-CRT IBI status and OS or DFS in this subgroup, except that a high post-CRT IBI status was significantly associated with poorer DFS (log-rank test: pre-CRT, OS, *p* = 0.06 and DFS, *p* = 0.34; post-CRT, OS, *p* = 0.21 and DFS, *p* = 0.03) (Figure 2a–d). Multivariable Cox proportional hazards regression analyses identified a high post-CRT IBI status as an independent predictor of poorer DFS in patients with ypN+ RC; however, no significant independent predictors of OS were identified in patients with ypN+ RC receiving CRT (Table 3 and Table 4). Furthermore, we evaluated the potential of the peri-CRT IBI status as a prognostic biomarker for identifying patients who might benefit from intensive postoperative chemotherapy, particularly among otherwise low-risk patients with RC without pathological lymph node metastasis (ypN−). Survival analyses demonstrated that both a high pre-CRT IBI and high post-CRT IBI were significantly associated with poorer OS and DFS than a low IBI in patients with ypN− RC (log-rank test: pre-CRT, OS, *p* < 0.001 and DFS, *p* < 0.001; post-CRT, OS, *p* = 0.001 and DFS, *p* = 0.002) (Figure 2e–h). Multivariable Cox proportional hazards regression analysis further identified a high pre-CRT IBI status as an independent prognostic factor for both OS and DFS in patients with ypN− RC (OS: HR, 6.74; 95% CI, 1.1–41.3; *p* = 0.04; DFS: HR, 7.68; 95% CI, 1.39–42.5; *p* = 0.02) (Table 5 and Table 6).

## 4. Discussion

Our study revealed several notable findings regarding the clinical significance of the peri-treatment IBI in patients with RC undergoing neoadjuvant CRT. First, the IBI measured before and after CRT was not significantly associated with most clinicopathological factors, suggesting that the IBI reflects the systemic inflammatory status not fully captured by conventional tumor-related indicators. Second, a high pre-CRT IBI was significantly associated with poorer OS and DFS and remained an independent prognostic factor in multivariable analyses. Finally, the IBI status was not a useful prognostic indicator in patients with ypN+ disease, except that a high post-CRT IBI was identified as an independent adverse prognostic factor for DFS. In contrast, a high pre-CRT IBI was an independent adverse prognostic factor for both OS and DFS in the ypN cohort. This finding may have greater clinical relevance for selecting patients for adjuvant therapy.

One of the major findings of this study is the prominent prognostic impact of the peri-treatment IBI, especially the pre-CRT IBI status, in patients with RC undergoing neoadjuvant CRT followed by curative resection. To understand the clinical significance of these findings, it is essential to elucidate the biological mechanisms underlying the IBI and its constituent parameters. The IBI is a novel, comprehensive inflammation-based biomarker calculated using the serum CRP level, neutrophil count, and lymphocyte count [14]. Each component reflects distinct yet interrelated pathways of the host immune-inflammatory response and tumor–host interactions. CRP is a classic acute-phase reactant synthesized by the liver in response to proinflammatory cytokines (most notably interleukin-6), which are frequently secreted by tumor cells or surrounding stromal cells within the tumor microenvironment [21]. Elevated CRP levels not only indicate a state of chronic systemic inflammation but also promote tumor proliferation, angiogenesis, and tissue remodeling, thereby facilitating a permissive environment for cancer progression and metastasis [22]. Simultaneously, circulating neutrophils and lymphocytes represent opposing arms of the host immune system during oncogenesis. Neutrophils play an active role in driving tumor progression through the secretion of various angiogenic factors and proteases, such as vascular endothelial growth factor and matrix metalloproteinases [23]. These factors actively remodel the extracellular matrix, enhance tumor cell motility, and promote neovascularization. Furthermore, neutrophils can suppress the cytolytic activity of T lymphocytes and natural killer cells, effectively shielding tumor cells from host immune surveillance [24]. Conversely, peripheral lymphocytes also play a critical role in the host cytotoxic immune response to tumors [17], and a depleted lymphocyte count, or lymphopenia, signifies impaired host immune surveillance and an inadequate capacity to mount an effective antitumor response [25]. Based on this evidence, combining these three parameters into a single index, the IBI, may provide a more accurate reflection of systemic inflammatory activity while accounting for host immunological exhaustion. Song et al. assessed preoperative IBI levels in 701 patients who underwent resection for hepatocellular carcinoma and showed that the IBI predicted poor prognosis after hepatectomy [12]. More recently, Zhao et al. evaluated the preoperative IBI status in 408 patients with esophageal squamous cell carcinoma who were assigned to either a primary or a validation cohort. They demonstrated the prognostic value of the IBI for survival outcomes in both cohorts [26]. Our research group also previously assessed the preoperative IBI status in 555 patients with CRC undergoing primary tumor resection and identified the preoperative IBI as a valuable predictive biomarker for perioperative risk and oncological outcomes [13]. However, that study focused on the clinical value of the preoperative IBI in patients with CRC, whereas the clinical relevance of the peri-treatment IBI during neoadjuvant CRT for predicting long-term oncological outcomes in patients with RC remains unclear. Taken together with our present findings, these previous studies suggest that the pre-CRT IBI may serve as a valuable prognostic indicator in patients with RC undergoing neoadjuvant CRT followed by curative resection.

Another major finding of this study is the clinical utility of the peri-treatment IBI stratified according to pathological nodal status (ypN) following neoadjuvant CRT. Subgroup analysis demonstrated that the prognostic value of the peri-treatment IBI differed according to the ypN status, with the pre-CRT IBI showing particularly strong prognostic performance in patients without pathological lymph node metastasis (ypN−). Although postoperative adjuvant chemotherapy is the standard of care for patients with pathological stage III or high-risk stage II CRC, its role in patients with locally advanced RC treated with neoadjuvant CRT remains uncertain, particularly among those without residual pathological lymph node metastasis (ypN−) [27]. These findings suggest that incorporating the peri-treatment IBI into postoperative risk stratification may identify patients who could benefit from intensified surveillance or adjuvant therapy, thereby enabling more individualized postoperative management of patients with ypN− RC. As a simple, inexpensive, and highly reproducible biomarker, the peri-treatment IBI—particularly when assessed before CRT—could help reduce undertreatment by flagging patients whose tumors appear low-risk on conventional pathology but are biologically aggressive.

In the present study, IBI was significantly higher in male patients than in female patients before and after CRT. This disparity between the sexes in systemic inflammation can largely be attributed to differences in body composition. Visceral adiposity, which is generally greater in men [28], stimulates the production of pro-inflammatory cytokines, including tumor necrosis factor-α and interleukin-6 [29]. This leads to increased inflammatory responses. Although the literature remains relatively limited, several prior reports have similarly documented higher systemic inflammatory responses in males [30,31].

An additional noteworthy observation is that the prognostic value of the pre-CRT IBI was particularly evident in the ypN− subgroup. Because CRT itself alters the systemic inflammatory and nutritional status, biomarkers measured after treatment may partly reflect treatment-induced changes rather than the patient’s baseline host condition. By contrast, the pre-CRT IBI is more likely to capture the intrinsic host inflammatory and nutritional status before therapeutic intervention, thereby providing prognostic information that complements the pathological response. Indeed, neither pre- nor post-CRT IBI was significantly correlated with pathological response metrics, such as tumor regression grade or pathological complete response. However, an elevated pre-CRT IBI was associated with subsequent distant metastasis, suggesting that baseline host-tumor inflammatory burden and malnutrition predispose patients to occult micrometastases. Conversely, an elevated post-CRT IBI was significantly associated with positive surgical margins and local recurrence, reflecting secondary systemic inflammation and persistent nutritional decline driven by inadequate local disease control. Taken together, evaluating dynamic changes in IBI before and after CRT might provide more comprehensive and sensitive prognostic insights, as demonstrated by our findings.

There are several potential limitations to this study. First, although we demonstrated several novel findings regarding pre- and post-CRT IBI status in patients with RC, the cohort size was relatively small. Due to this constraint, we were unable to perform comprehensive analyses, such as constructing predictive models or conducting incremental value analyses. Integrating IBI dynamics with other established clinicopathological variables, including clinical stage, imaging features, pathological response, vascular invasion, and tumor markers, in larger cohorts may more clearly determine whether this indicator enhances individualized risk stratification. Second, in addition to its retrospective design, patient enrollment spanned 15 years. Significant evolutions in clinical practice occurred during this time, including the widespread adoption of minimally invasive surgery and advances in novel molecular-targeted therapies for post-recurrence management. These evolutions may have acted as confounding factors influencing survival outcomes. Third, all patients were recruited from a single institution in Japan, which may limit the generalizability of our findings. Finally, although we focused on ypN status as an independent prognostic factor alongside IBI, exploratory stratifications yielded notable insights. Pre- or Post-CRT IBI showed pronounced prognostic value in male patients and the long-course CRT group, and retained significance for OS and DFS regardless of radiation response (Supplementary Table S2). Therefore, future large-scale, prospective validation across multiple institutions is necessary to overcome these limitations. Implementing uniform criteria for therapeutic regimens alongside long-term follow-up will substantiate our current results and solidify the role of pre- and post-CRT IBI status as robust prognostic and predictive biomarkers in RC.

## 5. Conclusions

Systematic assessment of the IBI before and after neoadjuvant CRT demonstrated its clinical utility and prognostic value in patients with RC undergoing curative resection. Our findings identify the peri-treatment IBI as a practical prognostic biomarker for oncological outcomes in this population. In particular, assessment of the IBI before CRT may provide valuable information for risk stratification and postoperative management, especially in patients without pathological lymph node metastasis.

## Figures and Tables

**Table 1:** Correlation between clinicopathological variables and Pre-/Post-CRT IBI status in Rectal Cancer patients.

		Pre-CRT IBI				Post-CRT IBI			
				High ^†^	Low ^†^			High ^†^	Low ^†^
Variable	*n*	Median ± IQR	*p* Value ^##^	(*n* = 21)	(*n* = 76)	Median ± IQR	*p* Value ^##^	(*n* = 16)	(*n* = 81)
Sex									
Male	70	0.23 ± 1.33	0.02 *	20	50	0.3 ± 0.75	0.04 *	14	56
Female	27	0.15 ± 0.2		1	26	0.18 ± 0.34		2	25
Age									
<64 (median) ^#^	46	0.2 ± 0.39	0.86	13	38	0.3 ± 0.89	0.42	10	36
>64	51	0.17 ± 0.64		8	38	0.24 ± 0.47		6	45
Clinical T category (cT)									
cT1/cT2	13	0.09 ± 0.12	0.051	0	13	0.24 ± 0.47	0.25	0	13
cT3/cT4	84	0.21 ± 0.64		21	63	0.28 ± 0.7		16	68
Clinical lymph node metastasis									
Positive	70	0.22 ± 0.55	0.17	16	54	0.26 ± 0.72	0.37	15	55
Negative	27	0.15 ± 0.25		5	22	0.29 ± 0.5		1	26
Clinical stage classification									
Stage I	11	0.1 ± 0.09	0.17	0	11	0.26 ± 0.41	0.5	0	11
Stage II	16	0.18 ± 0.8		5	11	0.32 ± 0.53		1	15
Stage III	70	0.22 ± 0.55		16	54	0.26 ± 0.72		15	55
Histological type									
Differentiated	83	0.18 ± 0.48	0.93	16	67	0.29 ± 0.6	0.44	13	70
Undifferentiated	14	0.28 ± 0.94		5	9	0.22 ± 0.17		3	11
Pathological T category (ypT)									
ypT1/ypT2	44	0.15 ± 0.28	0.07	7	37	0.26 ± 0.55	0.57	5	39
ypT3/ypT4	53	0.21 ± 0.99		14	39	0.3 ± 0.76		11	42
Venous invasion									
Positive	36	0.16 ± 0.44	0.65	8	28	0.26 ± 0.41	0.73	4	32
Negative	61	0.21 ± 0.57		13	48	0.27 ± 0.79		12	49
Lymphatic invasion									
Positive	48	0.16 ± 0.37	0.89	11	37	0.3 ± 0.7	0.73	9	39
Negative	49	0.22 ± 0.57		10	39	0.26 ± 0.56		7	42
Lymph node metastasis									
Positive	31	0.15 ± 0.41	0.36	6	25	0.22 ± 0.72	0.65	7	24
Negative	66	0.21 ± 0.56		15	51	0.28 ± 0.54		9	57
Type of radiation course									
Short course	44	0.17 ± 0.57	0.83	10	34	0.3 ± 0.57	0.81	6	38
Long course	53	0.2 ± 0.53		11	42	0.26 ± 0.64		10	43
Pathological effect of CRT									
Ineffective	63	0.2 ± 0.49	0.43	14	49	0.24 ± 0.67	0.76	10	53
Effective	34	0.16 ± 0.58		7	27	0.28 ± 0.53		6	28
Pathological complete response (pCR)									
Yes	6	0.1 ± 0.17	0.19	0	6	0.27 ± 0.12	0.92	0	6
No	91	0.2 ± 0.57		21	70	0.27 ± 0.67		16	75
UICC stage classification									
Stage0	6	0.1 ± 0.17	0.1	0	6	0.27 ± 0.12	0.79	0	6
Stage I	31	0.17 ± 0.3		5	26	0.26 ± 0.61		3	28
Stage II	29	0.26 ± 1.5		10	19	0.31 ± 0.83		6	23
Stage III	31	0.15 ± 0.41		6	25	0.22 ± 0.72		7	24
Resection margin status									
Positive	4	5.77 ± 9.53	0.09	3	1	2.67 ± 11.59	0.003 *	4	0
Negative	93	0.17 ± 0.43		18	75	0.26 ± 0.55		12	81
Local recurrence									
Yes	5	1 ± 10.42	0.34	3	2	1.86 ± 2.8	0.58	3	2
No	92	0.18 ± 0.46		18	74	0.26 ± 0.55		13	79
Distant recurrence									
Yes	13	0.48 ± 2.18	0.04 *	6	7	0.29 ± 1.44	0.63	4	9
No	84	0.16 ± 0.39		15	69	0.26 ± 0.56		12	72

* *p* < 0.05, ^#^ Median age at surgery is 64 years in this cohort. ^##^ Mann–Whitney U-test, ^†^ Cut-off thresholds of pre- and post-CRT IBI were determined by ROC analysis with Youden’s index for survival outcome in this cohort.

**Table 2:** Multivariate analysis for predictors of overall survival and disease-free survival.

**Overall Survival**						
	**Univariate**			**Multivariate**		
**Variable**	**HR**	**95% CI**	***p* Value**	**HR**	**95% CI**	***p* Value**
Sex, male	3.58	0.84–15.4	0.09	-	-	-
Age > 64 ^#^	1.28	0.55–2.97	0.56	-	-	-
Histological type, undifferentiated type	2.53	0.93–6.89	0.07	-	-	-
Pathological T category(ypT), ypT3/4	5.81	1.72–19.6	<0.01 *	2.12	0.53–8.39	0.29
Venous invasion, present	2.86	1.2–6.85	0.02 *	1.28	0.44–3.72	0.65
Lymphatic invasion, present	2.09	0.82–5.34	0.13	-	-	-
Lymph node metastasis, present	5.6	2.32–13.6	<0.01 *	4.76	1.72–13.2	<0.01 *
Pathological effect of CRT, ineffective	0.66	0.25–1.68	0.37	-	-	-
Pre-CRT IBI status, high ^†^	3.77	1.64–8.71	<0.01 *	3.43	1.42–8.27	<0.01 *
Post-CRT IBI status, high ^†^	3.92	1.66–9.24	<0.01 *	2.37	0.99–5.68	0.054
**Disease-Free Survival**						
	**Univariate**			**Multivariate**		
**Variable**	**HR**	**95% CI**	***p* Value**	**HR**	**95% CI**	***p* Value**
Sex, male	1.91	0.65–5.6	0.24	-	-	-
Age > 64 ^#^	1.07	0.48–2.41	0.89	-	-	-
Histological type, undifferentiated type	1.91	0.71–5.14	0.2	-	-	-
Pathological T category(ypT), ypT3/4	3.48	1.29–9.4	0.01 *	1.56	0.48–5.07	0.46
Venous invasion, present	2.3	1.01–5.25	0.048 *	1	0.34–2.91	0.99
Lymphatic invasion, present	1.77	0.75–4.18	0.19	-	-	-
Lymph node metastasis, present	5.53	2.33–13.1	<0.01 *	5.06	1.87–13.7	<0.01 *
Pathological effect of CRT, ineffective	0.58	0.23–1.47	0.23	-	-	-
Pre-CRT IBI status, high ^†^	3.28	1.43–7.5	0.049 *	2.73	1.13–6.62	0.03 *
Post-CRT IBI status, high ^†^	4.68	2.04–10.7	<0.01 *	3.14	1.33–7.4	<0.01 *

^#^ Median age at surgery is 64 years in this cohort, HR: hazard ratio, ^†^ Cut-off thresholds of pre- and post-CRT IBI were determined by ROC analysis with Youden’s index for survival outcome in this cohort. * *p* < 0.05.

**Table 3:** Multivariate analysis for predictors of overall survival in RC patients receiving CRT with pathological lymph node metastasis.

	Univariate			Multivariate		
Variable	HR	95% CI	*p* Value	HR	95% CI	*p* Value
Sex, male	2.29	0.51–10.34	0.28	-	-	-
Age > 64 ^#^	0.94	0.32–2.72	0.91	-	-	-
Histological type, undifferentiated type	2.01	0.55–7.36	0.29	-	-	-
Pathological category(ypT), ypT3/4	2.38	0.31–18.44	0.41	-	-	-
Venous invasion, present	1.43	0.44–4.66	0.55	-	-	-
Lymphatic invasion, present	0.8	0.25–2.63	0.72	-	-	-
Pathological effect of CRT, ineffective	0.39	0.09–1.76	0.22	-	-	-
Pre-CRT IBI status, high ^†^	2.85	0.93–8.71	0.07	-	-	-
Post-CRT IBI status, high ^†^	1.99	0.66–6.03	0.22	-	-	-

^#^ Median age at surgery is 64 years in this cohort. ^†^ Cut-off thresholds of pre- and post-CRT IBI were determined by ROC analysis with Youden’s index for survival outcome in this cohort. HR: hazard ratio.

**Table 4:** Multivariate analysis for predictors of disease-free survival in RC patients receiving CRT with pathological lymph node metastasis.

	Univariate			Multivariate		
Variable	HR	95% CI	*p* Value	HR	95% CI	*p* Value
Sex, male	2.99	0.68–13.27	0.15	-	-	-
Age > 64 ^#^	0.74	0.26–2.07	0.56	-	-	-
Histological type, undifferentiated type	1	0.22–4.45	1	-	-	-
Pathological category(ypT), ypT3/4	3.33	0.43–25.44	0.25	-	-	-
Venous invasion, present	1.84	0.58–5.82	0.30	-	-	-
Lymphatic invasion, present	1.3	0.41–4.18	0.65	-	-	-
Pathological effect of CRT, ineffective	0.31	0.07–1.4	0.13	-	-	-
Pre-CRT IBI status, high ^†^	2.83	1.01–7.94	0.048 *	2.69	0.95–7.67	0.06
Post-CRT IBI status, high ^†^	3.11	1.07–8.99	0.04 *	2.92	1.01–8.48	0.049 *

^#^ Median age at surgery is 64 years in this cohort. ^†^ Cut-off thresholds of pre- and post-CRT IBI were determined by ROC analysis with Youden’s index for survival outcome in this cohort. HR: hazard ratio, * *p* < 0.05.

**Table 5:** Multivariate Analysis for Predictors of Overall Survival in RC patients receiving CRT without pathological lymph node metastasis.

	Univariate			Multivariate		
Variable	HR	95% CI	*p* Value	HR	95% CI	*p* Value
Sex, male	-	-	0.96	-	-	-
Age > 64 ^#^	3.09	0.62–15.3	0.17	-	-	-
Histological type, undifferentiated type	3.14	0.63–15.7	0.16	-	-	-
Pathological T category(ypT), ypT3/4	4.26	0.86–21.1	0.08	-	-	-
Venous invasion, present	2.18	0.54–8.82	0.28	-	-	-
Lymphatic invasion, present	2.9	0.58–14.5	0.19	-	-	-
Pathological effect of CRT, ineffective	1.6	0.39–6.25	0.53	-	-	-
Pre-CRT IBI status, high ^†^	10.4	2.09–51.7	<0.01 *	6.74	1.1–41.3	0.04 *
Post-CRT IBI status, high ^†^	7.45	1.82–30.4	<0.01 *	2.61	0.54–12.7	0.23

^#^ Median age at surgery is 64 years in this cohort. ^†^ Cut-off thresholds of pre- and post-CRT IBI were determined by ROC analysis with Youden’s index for survival outcome in this cohort. HR: hazard ratio, * *p* < 0.05.

**Table 6:** Multivariate Analysis for Predictors of Disease-Free Survival in RC patients receiving CRT without pathological lymph node metastasis.

	Univariate			Multivariate		
Variable	HR	95% CI	*p* Value	HR	95% CI	*p* Value
Sex, male	1.1	0.22–5.47	0.9	-	-	-
Age > 64 ^#^	2.81	0.57–13.9	0.21	-	-	-
Histological type, undifferentiated type	4.37	1.04–18.3	0.04 *	2.34	0.54–10.1	0.26
Pathological T category(ypT), ypT3/4	1.45	0.36–5.8	0.6	-	-	-
Venous invasion, present	0.88	0.18–4.38	0.88	-	-	-
Lymphatic invasion, present	1.2	0.3–4.79	0.8	-	-	-
Pathological effect of CRT, ineffective	1.5	0.38–6	0.57	-	-	-
Pre-CRT IBI status, high ^†^	12.2	2.45–60.7	<0.01 *	7.68	1.39–42.5	0.02 *
Post-CRT IBI status, high ^†^	6.53	1.63–26.2	<0.01 *	2.59	0.59–11.3	0.21

^#^ Median age at surgery is 64 years in this cohort. ^†^ Cut-off thresholds of pre- and post-CRT IBI were determined by ROC analysis with Youden’s index for survival outcome in this cohort. HR: hazard ratio, * *p* < 0.05.

## Data Availability

The data presented in this study are available from the corresponding author upon reasonable request.

## Supplementary Materials

The following supporting information can be downloaded at: https://www.mdpi.com/article/10.3390/cancers18172784/s1, Figure S1: Prognostic impact of dynamic change in pre- and post-CRT IBI status in patients with RC undergoing curative surgery after preoperative CRT; Table S1: Multivariate Analysis for Predictors of Overall Survival and Disease-Free Survival; Table S2: Subgroup analysis of the prognostic significance of pre- and post-CRT IBI for Overall Survival and Disease-Free Survival.
