# Supplementary material for: Prognostic Significance of Peri-Treatment Inflammatory Burden Index in Patients with Rectal Cancer Undergoing Preoperative Chemoradiotherapy
Source: Cancers (Basel). 2026 Aug 27;18(17):2784. doi: 10.3390/cancers18172784 (PMC13564710; doi:10.3390/cancers18172784)
Supplement: Supplementary file 1 [file cancers-18-02784-s001.zip › cancers-4486504-supplementary.pdf]

SUPPLEMENTARY FIGURE AND TABLE

Prognostic Significance of Peri-Treatment Inflammatory Burden Index in Patients with Rectal Cancer Undergoing Preoperative Chemoradiotherapy

Takanobu Mori [1,2], Yoshinaga Okugawa [2,3,*], Tadanobu Shimura [2], Ma Ruiya [2], Naru Mizuno [2], Shinji Yamashita [2], Akira Yamamoto [2], Takahito Kitajima [2,3], Hiroki Imaoka [2], Mikio Kawamura [2], Yuhki Koike [2], Hiromi Yasuda [2], Yoshiki Okita [2], Shigeyuki Yoshiyama [2], Minako Kobayashi [2], Tomofumi Koide [1], Masaki Ohi [2] and Yuji Toiyama [2,*]

1 Department of Clinical Nutrition, Mie University Hospital, Mie, Japan
2 Department of Gastrointestinal and Pediatric Surgery, Division of Reparative Medicine, Institute of Life Sciences, Mie University Graduate School of Medicine, Mie, Japan
3 Department of Genomic Medicine, Mie University Hospital, Mie, Japan
* Correspondence: Yuji Toiyama and Yoshinaga Okugawa; Department of Gastrointestinal and Pediatric Surgery, Division of Reparative Medicine, Institute of Life Sciences, Mie University Graduate School of Medicine, Mie, Japan; Telephone: +81-59-231-5274; Y. Toiyama: ytoi0725@med.mie-u.ac.jp, Y. Okugawa: yosinaga@med.mie-u.ac.jp (https://orcid.org/0000-0002-0417-5559)

Table S1. Multivariate Analysis for Predictors of Overall Survival and Disease-Free Survival

**Overall Survival**						
	Univariate			Multivariate		
Variable	HR	95% CI	*P* Value	HR	95% CI	*P* Value
Sex, male	3.58	0.84–15.4	0.09	-	-	-
Age>64[#]	1.28	0.55–2.97	0.56	-	-	-
Histological type, undifferentiated type	2.53	0.93–6.89	0.07	-	-	-
Pathological T category(ypT),ypT3/4	5.81	1.72–19.6	**<0.01***	2.17	0.56–8.47	0.27
Venous invasion, present	2.86	1.2–6.85	**0.02***	1.44	0.52–3.97	0.49
Lymphatic invasion, present	2.09	0.82–5.34	0.13	-	-	-
Lymph node metastasis, present	5.6	2.32–13.6	**<0.01***	6.01	2.14–16.92	**<0.01***
Pathological effect of CRT, ineffective	0.66	0.25–1.68	0.37	-	-	-
Pre-/post-CRT IBI status, High†-High†	5.41	2.2–13.32	**<0.01***	8.32	3.04–22.78	**<0.01***
**Disease-Free Survival**						
	Univariate			Multivariate		
Variable	HR	95% CI	*P* Value	HR	95% CI	*P* Value
Sex, male	1.91	0.65–5.6	0.24	-	-	-
Age>64[#]	1.07	0.48–2.41	0.89	-	-	-
Histological type, undifferentiated type	1.91	0.71–5.14	0.2	-	-	-
Pathological T category(ypT),ypT3/4	3.48	1.29–9.4	**0.01***	1.40	0.43–4.54	0.58
Venous invasion, present	2.3	1.01–5.25	**0.048***	1.18	0.43–3.25	0.74
Lymphatic invasion, present	1.77	0.75–4.18	0.19	-	-	-
Lymph node metastasis, present	5.53	2.33–13.1	**<0.01***	5.59	2.12–14.71	**<0.01***
Pathological effect of CRT, ineffective	0.58	0.23–1.47	0.23	-	-	-
Pre-/post-CRT IBI status, High†-High†	6.81	2.78–16.73	**<0.01***	8.29	3.23–21.29	**<0.01***

[#]Median age at surgery is 64 years in this cohort.HR: hazard ratio, *p<0.05, †Cut-off thresholds of pre- and post-CRT IBI were determined by ROC analysis with Youden's index for survival outcome in this cohort.

Table S2. Subgroup analysis of the prognostic significance of pre- and post-CRT IBI for Overall Survival and Disease-Free Survival

Variable	n	Pre-CRT IBI Low/High † (n)	Pre-CRT IBI HR (95% CI)	*P* value	Post-CRT IBI Low/High † (n)	Post-CRT IBI HR (95% CI)	*P* value
**Overall Survival**							
Sex							
Male	70	50/20	3.99 (1.50–10.68)	**<0.01***	56/14	5.81 (1.87–17.98)	**<0.01***
Female	27	26/1	NE	0.84	25/2	NE	0.76
Age							
<64 years#	46	38/8	5.38 (1.02–28.48)	**0.048***	36/10	10.80 (2.03–57.34)	**<0.01***
≥64 years#	51	38/13	5.72 (1.56–21.06)	**0.01***	45/6	5.56 (1.05–29.32)	**0.04***
Radiotherapy regimen							
Short course	44	34/10	2.42 (0.58–10.06)	0.22	38/6	3.23 (0.58–18.01)	0.18
Long course	53	42/11	16.51 (3.24–84.09)	**<0.01***	43/10	11.64 (2.43–55.85)	**<0.01***
Pathological effect of CRT (JSCCR grade)							
Grade 0–1	63	49/14	4.11 (1.22–13.80)	**0.02***	53/10	6.36 (1.58–25.60)	**<0.01***
Grade 2–3	34	27/7	9.28 (1.46–59.01)	**0.02***	28/6	13.09 (1.45–118.18)	**0.02***

Variable	n	Pre-CRT IBI Low/High † (n)	Pre-CRT IBI HR (95% CI)	*P* value	Post-CRT IBI Low/High † (n)	Post-CRT IBI HR (95% CI)	*P* value
**Disease-Free Survival**							
Sex							
Male	70	50/20	4.42 (1.56–12.56)	**<0.01***	56/14	14.55 (4.29–49.32)	**<0.01***
Female	27	26/1	NE	0.68	25/2	NE	0.55
Age							
<64 years#	46	38/8	4.82 (0.94–24.61)	0.06	36/10	6.78 (1.43–32.06)	**0.02**
≥64 years#	51	38/13	4.86 (1.25–18.93)	**0.02**	45/6	20.42 (3.43–121.69)	**<0.01***
Radiotherapy regimen							
Short course	44	34/10	1.26 (0.31–5.05)	0.74	38/6	7.04 (1.25–39.51)	**0.03**
Long course	53	42/11	27.27 (5.65–131.69)	**<0.01***	43/10	13.87 (2.89–66.49)	**<0.01***
Pathological effect of CRT (JSCCR grade)							
Grade 0–1	63	49/14	3.00 (0.88–10.19)	0.08	53/10	13.08 (3.13–54.57)	**<0.01***
Grade 2–3	34	27/7	16.01 (2.23–114.83)	**<0.01***	28/6	14.66 (1.56–137.74)	**0.02**

#Median age at surgery is 64 years in this cohort. HR: hazard ratio. *p<0.05. †Cut-off thresholds of pre- and post-CRT IBI were determined by ROC analysis with Youden's index for survival outcome in this cohort.

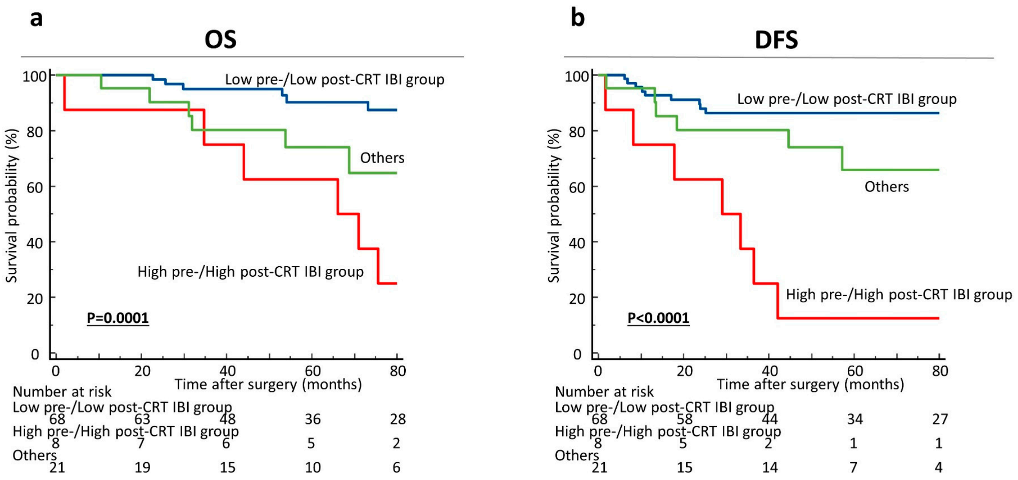

Figure S1. Prognostic impact of dynamic change in pre- and post-CRT IBI status in patients with RC undergoing curative surgery after preoperative CRT.
